# Dynamic and static functional network connectivity distinguish symptomatic and non‐symptomatic individuals with CADASIL

**DOI:** 10.1002/alz.093984

**Published:** 2025-01-09

**Authors:** Henry J Bockholt, Jane S Paulsen, Bradley T Baker, Helen Petropoulos, Arvind Caprihan, Kevin M. Johnson, Michael D Geschwind, Laura B. Eisenmenger, David S Liebeskind, Jordan Clemsen, Nicholas Pasley, William H. Adams, Michael A Newton, Vince D Calhoun

**Affiliations:** ^1^ Tri‐Institutional Center for Translational Research in Neuroimaging and Data Science (TReNDS), Atlanta, GA USA; ^2^ University of Wisconsin‐Madison, Madison, WI USA; ^3^ The Mind Research Network, Albuquerque, NM USA; ^4^ School of Medicine and Public Health, University of Wisconsin‐Madison, Madison, WI USA; ^5^ University of California, San Francisco (UCSF), San Francisco, CA USA; ^6^ University of California‐Los Angeles, Los Angeles, CA USA; ^7^ Loyola University Chicago, Chicago, IL USA

## Abstract

**Background:**

This study focuses on Cerebral Autosomal Dominant Arteriopathy with Subcortical Infarcts and Leukoencephalopathy (CADASIL), a key model for studying arterial degradation and its impact on brain network communication. We explore functional network connectivity in CADASIL patients, shedding light on how arterial changes affect brain network interactions.

**Method:**

Overcoming COVID‐19 challenges, we've enrolled over 200 participants for longitudinal assessments. The study compares Symptomatic (SYM) individuals (Rankin Scale scores 1‐3) with Non‐Symptomatic (NSYM) counterparts. The SYM group, mainly females (63) and Caucasians (101), shows lower cognitive performance (average MOCA score 25.2) and processing speed (SDMT average 43.7) compared to NSYM's higher scores (MOCA 26.8, SDMT 51.4). Additionally, the SYM group, older on average (53 years), exhibits more functional impairment (average WHODAS 9.1) than NSYM (47.7 years, WHODAS 3.1). Advanced neuroimaging (3T Siemens Prisma Fit or 3T Signa Premier scanners) and Spatially Constrained Independent Component Analysis are used for dynamic functional network connectivity (dFNC) analysis.

**Results:**

The SYM and NSYM groups display significant cognitive and functional differences. Connectivity analyses reveal moderate to substantial differences in both static and dynamic states, particularly in Visual and Cognitive Control domains. Static analysis identifies disparities in 8 intrinsic connectivity networks across 5 domains, while dynamic analysis shows 3 of 4 states with significant differences in cognitive control networks.

**Conclusion:**

This research underlines the profound impact of CADASIL on brain network connectivity, notably in cognitive control and visual processing. The findings, integral to the USA CADASIL Consortium, aim to characterize CADASIL's clinical and biological markers. These results are pivotal for global efforts to understand and treat vascular contributions to cognitive impairment and dementia, enhancing the landscape of CADASIL research and therapy development.

